# Commentary: Language teacher motivation, autonomy and development in East Asia

**DOI:** 10.3389/fpsyg.2022.1018850

**Published:** 2022-09-20

**Authors:** Fang Su, Lintao Zhang

**Affiliations:** ^1^School of English Studies, Research Center for Linguistics and Applied Linguistics, Xi'an International Studies University, Xi'an, China; ^2^Graduate School of Education, Beijing Foreign Studies University, Beijing, China

**Keywords:** language teacher, autonomy, motivation, development, East Asia

We are living in challenging times, especially when the COVID-19 dramatically changed the way of learning and teaching. Under this circumstance, the motivation and autonomy of teachers are pivotal. Motivation is a want toward future action (Ryan and Deci, 2002), while autonomy is the capacity for self-directed professional action, development and freedom from control by others over them (Smith, 2003). Both motivation and autonomy are critical components of genuinely successful teachers in the process of their professional development.

Regarding teacher motivation, autonomy and development in the second language (L2) context as an interwoven phenomenon, *Language teacher motivation, autonomy and development in East Asia*, a newly published volume, edited by Yuzo Kimura, Luxin Yang, Tae-Young Kim and Yoshiyuki Nakata, displays the latest theoretical and empirical local studies of teacher motivation, autonomy and development. As researchers of teacher development and front-line language teachers, we have found this volume insightful, stimulating and engaging and see it as a reflective resource for personal development.

Beginning with an introductory chapter (Chapter 1), the volume consists of two main parts: the theoretical perspectives (Chapters 2–4) and the empirical studies (Chapters 6–11), wrapped up by a commentary chapter (Chapter 12). In the introductory chapter, the four editors introduced the theme, significance, and the educational contexts in East Asia, providing readers with basic contextual understanding of issues under investigation.

Shedding light on the theoretical perspectives, the first part of the book introduces the theoretical frameworks in studying teacher motivation, autonomy and development and serves as the theoretical underpinnings for subsequent empirical studies. It begins with Hiver's introduction to complexity theory and its utility as a conceptual framework for researching the lives and work of language teachers. Hiver sees complexity theory as a meta-theory which can inform transdisciplinary research in the field and “assist in plugging gaps left by conventional research paradigms” (Kimura et al., 2022, p. 15). In Chapter 3, Nakata integrates the notions of self-regulation and motivation in his discussion of the social aspect of teacher autonomy in the classroom. He concludes that teacher autonomy is likely to be promoted in a mode of socially shared regulation. Zhang, in Chapter 4, provides a thorough review of the theoretical paradigm shifts in second language teacher education and development. She points out that the pivotal of the theoretical shifts is epistemological around the nature of knowledge. Her review of theoretical shifts covers behavioristic, cognitive, constructivism, sociocultural approaches, and complex dynamic systems theory (CDST), charting the theoretical road map for informing teacher development research.

The second part, including seven chapters, dives into the empirical perspectives of teacher motivation, autonomy and development. In Chapters 5 and 7, Sampson and Kimura take an ethnographic approach and visualize the trajectory of teachers' motivation from a CDST perspective. These two chapters illustrate how teacher motivation or autonomy can be described through CDST. Looking into the initial career motives and demotivating factors of 144 secondary EFL teachers quantitatively, Kim and Kim in chapter 6 conclude that teachers' passion, expected emotional rewards, socioeconomic considerations drive them to enter the teaching profession, while too much paper work and a lack of teaching autonomy demotivate them on the job. In chapter 8, Qian investigates teacher autonomy through a longitudinal case study guided by grounded theory, and classifies it into three levels.

In Chapter 9, Sakui and Cowie examine their experiences of joining a community of practice (CoP) of digital technology and conclude that it is vital to reach out across multiple communities to encourage wider base for professional learning. Similarly, through a CoP theoretical lens, Yang in Chapter 10 investigates the changes of three EFL teachers' teaching beliefs and practice in group lesson discussions. She finds that participating in group lesson discussions helps teachers gain new understanding of language teaching and learning and improve the quality of their teaching practice. Kim in Chapter 11 examines the formation of a secondary school pre-service teacher's concept regarding reading in a teacher education course from a sociocultural theory (SCT) perspective. It is discovered that the participant's concept regarding reading developed from everyday concept to academic concept and finally formed a true concept during this course. In the last commentary chapter, Kubanyiova sets the scene of teacher development research in a post-COVID world and calls for telling multiple stories of language teachers' lives with collaborative visions for future study.

This book provides the depth and breadth of research in teacher motivation, autonomy and development in East Asia both theoretically and methodologically. Theoretically, it not only charts a theoretical road map for the study of language teacher motivation, autonomy and development, but also illustrates how each theory can be adopted to explain the real life stories of language teachers. Drawing from theoretical frameworks around CDST, CoP, and socially shared regulation process, this book threw light on the complex, dynamic and changing nature of teacher development, providing insightful understanding of teacher's lived experiences in local context. What's more, it indicates that CDST seems to hold great promises. CDST is “a theory of change, evolution, adaptation and development” (Morrison, 2008, p. 16), which suits well in understanding and advancing the complex, dynamic and socially mediated processes of language teacher development.

Methodologically, this book illustrates well how different research methods/approaches such as auto-ethnography, quasi-ethnography, survey and case study can be applied in researching language teacher motivation, autonomy and development. Chapters 5, 7, 8, 10, and 11 show the use of (auto/quasi) ethnography, while chapter 6 investigates teacher motivation through a survey, and chapter 8 adopted a case study approach. In each of these chapters, the author(s) presents clearly the design of the study such as the participant selection process, data collection and analysis procedure, which are good samples to learn from for front line teachers, especially those green-hand researchers.

Pedagogically, this volume highlights the connection of the lives of both teachers and students in researching teacher motivation, autonomy and development. For example, Nakata's proposed concept of socially shared regulation brings students into the scene and suggests a strong reciprocal relationship between learner autonomy and teacher autonomy. In the same vein, Zhang stresses the link between teacher and student learning in doing teacher development research. Particularly, Sampson highlights the emotional impact of his students on his motivational trajectories. Besides, connection between learners' linguistic practice and teachers' experiences are also illustrated in Qian's and Kim's empirical studies.

To sum up, this book is stimulating in that the stories may resonate with the lived experiences of language teachers in a more general global context. Thus, it serves as a timely “reflective resource” (Kimura et al., 2022, p. 2) for language teachers socializing into a world that has been dramatically affected by the COVID-19 pandemic. It provides a contextual foundation for either front-line teachers or teacher researchers with an extensive account of theoretical and empirical perspectives in studying teacher motivation, autonomy and development. In addition, this book focuses on the complex, dynamic and socially mediated processes of language teacher development from a participant-centered point of view with the connection of students' lived experiences. This makes the book enlightening for language teachers in response to the ever-challenging local and global context in a post-COVID era.

However, the book would be more comprehensive and holistic if it had further explained how language teacher motivation, autonomy and development interact with each other, and how it can be understood from different perspective as an interwoven phenomenon. This, at the same time, may leave a research space for further exploration.

## Author contributions

FS drafted the General Commentary. LZ did the revision for the text. All authors contributed to the article and approved the submitted version.

## Conflict of interest

The authors declare that the research was conducted in the absence of any commercial or financial relationships that could be construed as a potential conflict of interest.

## Publisher's note

All claims expressed in this article are solely those of the authors and do not necessarily represent those of their affiliated organizations, or those of the publisher, the editors and the reviewers. Any product that may be evaluated in this article, or claim that may be made by its manufacturer, is not guaranteed or endorsed by the publisher.
